# Dynamic and Static Assistive Strategies for a Tailored Occupational Back-Support Exoskeleton: Assessment on Real Tasks Carried Out by Railway Workers

**DOI:** 10.3390/bioengineering11020172

**Published:** 2024-02-10

**Authors:** Christian Di Natali, Tommaso Poliero, Vasco Fanti, Matteo Sposito, Darwin G. Caldwell

**Affiliations:** 1 Department of Advanced Robotics, Istituto Italiano di Tecnologia, Via San Quirico 19d, 16163 Genoa, Italy; tommaso.poliero@iit.it (T.P.); vasco.fanti@iit.it (V.F.); matteo.sposito@iit.it (M.S.); darwin.caldwell@iit.it (D.G.C.); 2 Department of Informatics, Bioengineering, Robotics and Systems Engineering (DIBRIS), Universita’ degli Studi di Genova (UniGe), 16145 Genova, Italy

**Keywords:** industrial exoskeleton, back-support exoskeleton, assistive control, gravity compensation, real working task

## Abstract

This study on occupational back-support exoskeletons performs a laboratory evaluation of realistic tasks with expert workers from the railway sector. Workers performed both a static task and a dynamic task, each involving manual material handling (MMH) and manipulating loads of 20 kg, in three conditions: without an exoskeleton, with a commercially available passive exoskeleton (Laevo v2.56), and with the StreamEXO, an active back-support exoskeleton developed by our institute. Two control strategies were defined, one for dynamic tasks and one for static tasks, with the latter determining the upper body’s gravity compensation through the Model-based Gravity Compensation (MB-Grav) approach. This work presents a comparative assessment of the performance of active back support exoskeletons versus passive exoskeletons when trialled in relevant and realistic tasks. After a lab characterization of the MB-Grav strategy, the experimental assessment compared two back-support exoskeletons, one active and one passive. The results showed that while both devices were able to reduce back muscle activation, the benefits of the active device were triple those of the passive system regarding back muscle activation (26% and 33% against 9% and 11%, respectively), while the passive exoskeleton hindered trunk mobility more than the active mechanism.

## 1. Introduction

Exoskeletons are wearable devices that generate forces/torques on one or multiple human joints to support the execution of physical activities [1]. If the wearer is a worker, if the considered activity is work-related, and if the assisted joint is the back, exoskeletons are referred to as occupational Back Support Exoskeletons (oBSEs), [2]. Laboratory studies have documented how oBSEs can effectively reduce back muscle activation, with the associated potential to reduce back injuries [3,4,5,6,7].These benefits can be linked with potential ergonomic and productivity improvements [8,9], and new methods are being developed to update classical ergonomic risk evaluation tools (e.g, the NIOSH Lifting Index, [10]) to include the benefits linked with oBSE usage [11,12]. To provide assistance, oBSEs can rely either on purely mechanical elements or on active actuators. The former are called passive exoskeletons, while the latter are referred to as active exoskeletons. To increase the chances of successful adoption of exoskeletons in the workplace, it is important to focus on exoskeleton versatility. In this context, versatility refers to the exoskeleton’s ability to adapt to the various activities that can be found in uncontrolled (real-world) scenarios. While passive devices are less versatile by design, active ones can rely on sensors and algorithms to adapt the assistive output to the demands of the task [13,14,15]. Currently, occupational back support exoskeletons are entering a new development stage in which evaluation of their performance is now expected to take place in field studies [16,17,18]. These studies aim to highlight and verify the potential of exoskeletons to perform as promised by reducing overload and being accepted by workers within industrial environments. However, further work is needed to enhance comfort, exoskeleton–task fit, and user acceptance.

The work presented in this paper describes one of the evaluation steps in a multistage experimental study [19]. This work presents a laboratory-based exoskeleton performance evaluation of a dedicated active oBSE. This oBSE was created within the STREAM project (www.streams2r.eu (accessed on 20 December 2023)) to address the demands of railway workers in their MMH (manual material handling)-based tasks. A comparison is made between the custom-made active exoskeleton (StreamEXO) and a state-of-the-art general-purpose passive device (Laevo) in areas involving exoskeleton–task fit and the reduction of muscular activity. Testing involved ten railway workers who closely simulated their daily activities in a laboratory setting, and a comparison conducted of their performance with and without the exoskeleton was made. De Bock et al. [20] have already highlighted that lab testing and real-world tests may provide different results. However, testing conducted on a work site may suffer from interference with high-precision measurement equipment (e.g., gyroscopes and EMG) [21,22,23]. Thus, a key aspect of laboratory testing is how faithful the worker’s movement in the laboratory is compared to the real world. To ensure maximum fidelity, ergonomics studies were undertaken to ensure the accuracy of the reproduction of the task in the lab, and this was closely coupled to several worksite inspections. Moreover, experienced workers were involved in defining and executing tasks with which they were familiar.

**Figure 1:**
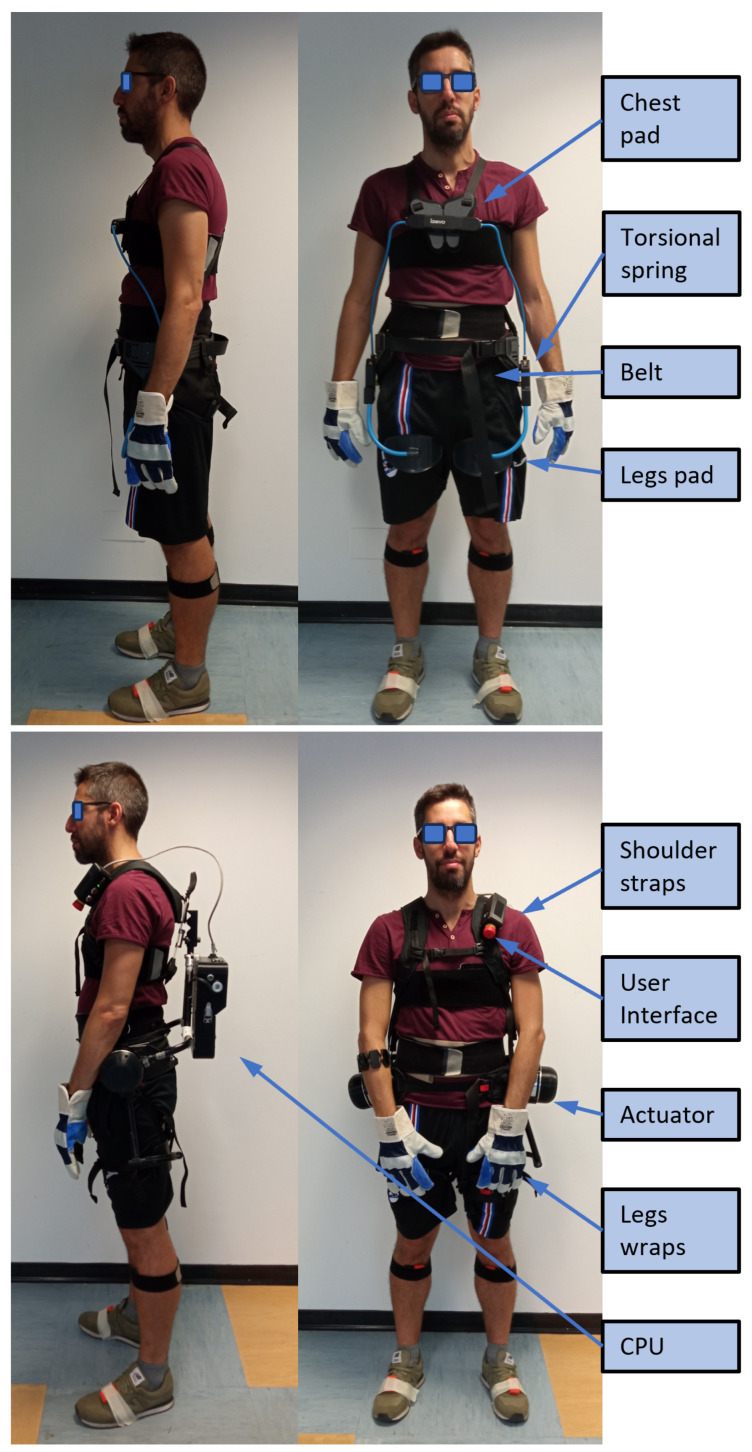
A comparison of the Laevo v2.56 and StreamEXO worn by the railway workers.

The workers were asked to perform two main sub-tasks, which were selected to be the most characteristic and impactful with respect to the daily work of track-side electric cable renewal [19]. These sub-tasks have different characteristics, being classified as dynamic and static, respectively. To allow comparison between different exoskeletons, as in [24,25,26], each task was performed under three conditions (Figure 1): (a) without an exoskeleton, (b) with a commercially available passive exoskeleton, the Laevo v2.56 (Laevo, Rijswijk, The Netherlands), and (c) with the StreamEXO, an active custom-made back-support exoskeleton developed by Istituto Italiano di Tecnologia. More details on the two BSEs used in this study are presented in Section 2.1, along with a description of the specific assistance strategies for active oBSEs specifically defined for dynamic and static activities. In particular, for the first time, this work presents a static strategy (and its characterization) that aims to compensate for the gravity of the upper body of workers during static tasks. The motivation for this new strategy arises from the limitations identified in [26]. Section 2 outlines the experimental protocol, while Section 3) outlines the data collection (Section 3.1), data processing (Section 3.2), and statistical analysis. Presentation of the results (Section 4) and a discussion (Section 5) are followed by the conclusions (Section 6).

## 2. Materials and Methods

### 2.1. Exoskeleton Comparison: Laevo v2.56 and StreamEXO

The StreamEXO active back support exoskeleton has been designed and tailored to the requirements of the railway industry, involving activities such as using tools. The workers wear it only when performing manual material handling or adopting incongruous posture. Among the main desired features are (i) Ingress Protection (IP) rating for outdoor usage, (ii) batteries that guarantee continuous operation, (iii) use of buckles and straps to promote quick and intuitive donning/doffing, and (iv) enhanced freedom of movement to reduce hindrance. The StreamEXO is a one-size-fits-all exoskeleton design that houses two electrical motors and a backpack integrating custom electronics, sensors, and a battery (see Figure 1). It has an overall weight of 7.5 kg. The motors are torque-controlled and the assistive strategy varies according to the task, e.g., static or dynamic.

In the dynamic condition, the assistive strategy is based on a well known algorithm presented in [3], which has been modulated to generate higher assistive forces depending on the workers’ physique and the nature of the heavy activities being carried out. In particular, the exoskeleton torque $\tau_{exo}$ is a function of the handled load and the trunk inclination in the sagittal plane (θ), as in (1):

(1) $$\tau_{exo}=min\left(\tau_{max},k_{imu}sin\left(\theta -\theta_{0}\right)+k_{emg}\gamma \right)$$

where $\tau_{max}=50$, $k_{imu}=20$, $k_{emg}=32$, $\theta_{0}=15$, and γ=1 whenever a load is handled and γ=0 otherwise. Moreover, the assistive torque is bounded in the $\left[0,\tau_{max}\right]$ interval.

**Figure 2:**
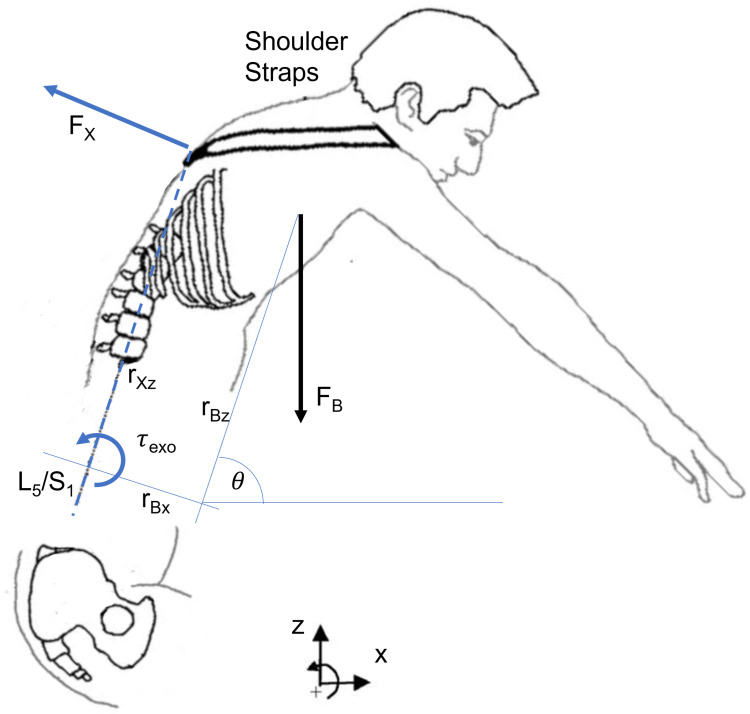
The biomechanical model and the applied forces and loads.

The static assistive strategy was specifically designed to improve the user experience and performance of the current torque-controlled active exoskeleton [26]. The StreamEXO implements a solution to compensate for the gravity effect of the upper body weight (UBW) based on the biomechanical model presented in [11] and shown in Figure 2. The following formulation aims to correlate $\tau_{exo}$ with the user’s anthropometric data to balance the user’s UBW against gravity. $\tau_{exo}$ is parameterized as a function of the user’s anthropometric data (body weight BW and body height BH) and trunk inclination (θ). Defining $\tau_{L5/S1}$ as the torque that the back muscles need to generate to balance the UBW, it holds that

(2) $$\begin{array}{c} \begin{array}{cc} \tau_{L5/S1}= & F_{B}r_{Bx}cos\left(\theta +arctan\left(\frac{r_{Bx}}{r_{Bz}}\right)\right)+\\ \; & F_{B}r_{Bz}sin\left(\theta +arctan\left(\frac{r_{Bx}}{r_{Bz}}\right)\right), \end{array} \end{array}$$

where the UBW-gravity force $F_{B}$ is applied in $r_{B}=\left[r_{Bx},\;r_{Bz}\right]$, assuming the origin of the Cartesian space to be the application point of the compressing force of the intervertebral discs ($F_{L5/S1}$).

For static activities, we considered the following relationship between the exoskeleton force $F_{X}$, torques $\tau_{exo}$, and application point $r_{Xz}$ with the torque on the spine joint $\tau_{L5/S1}$. Therefore, we make the following assumption:

(3) $$F_{X}r_{Xz}=\tau_{exo}=\tau_{L5/S1}.$$

Additionally, from [27,28,29] it holds that

(4) $$\begin{array}{c} \begin{array}{cc} r_{Bx}= & 0.012BH\\ r_{Bz}= & 0.18BH\\ F_{B}= & 60.14\%BW9.81\;{m/s}^{2}. \end{array} \end{array}$$

It follows that

(5) $$\begin{array}{c} \begin{array}{cc} \tau_{exo}= & \alpha [r_{Bx}cos\left(\theta +arctan\left(\frac{r_{Bx}}{r_{Bz}}\right)\right)+\\ \; & r_{Bz}sin\left(\theta +arctan\left(\frac{r_{Bx}}{r_{Bz}}\right)\right)]F_{B}/r_{Xz} \end{array} \end{array}$$

and, eventually,

(6) $$\tau_{exo}=\delta BWBH\left(0.07cos\left(\theta +0.067\right)+1.06sin\left(\theta +0.067\right)\right),$$

where $\delta =\alpha /r_{Xz}$ (considering $r_{Xz}=0.25$ m) and α=0.5 (as the total torque is provided by the exoskeleton’s two motors). In this study, we set BW=85 and BH=175. This provides all workers with the same assistance and allows for comparison of the maximum assistance provided by the two oBSEs in the static condition.

**Figure 3:**
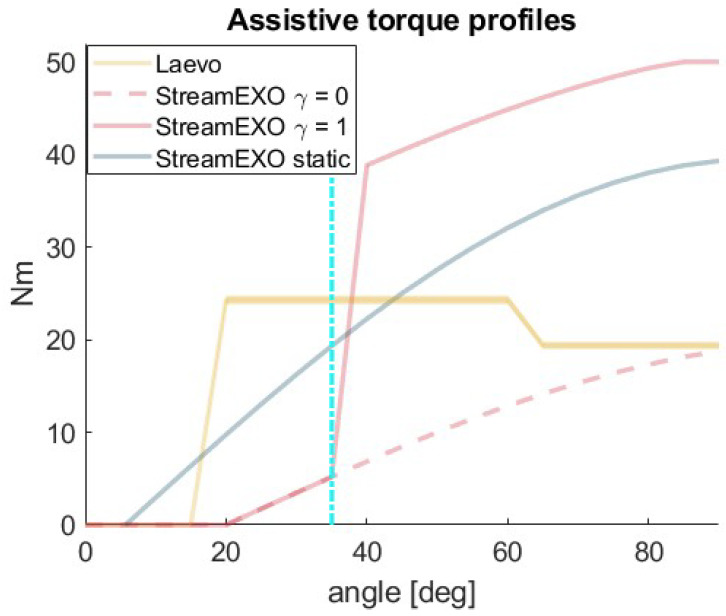
The total assistive torques provided by the StreamEXO and Laevo v2.56 as a function of the trunk angle. The solid red line corresponds to situations where the StreamEXO user is handling a load (γ=1), while the profile associated with the dashed red line corresponds to those tasks where no load is handled (γ=0). The solid yellow line with the shaded contour represents the result for the Laevo v2.56 presented in [26]. The blue solid line represents the assistance provided by the StreamEXO in the static task. A vertical dash-dotted cyan line is used to identify a possible instant when the worker grasps an external load and γ turns to 1.

The second oBSE used in this study was the Laevo v2.56, which is a passive 3.0 kg commercial device that delivers torques by means of gas springs. Its maximum claimed torque assistance is 40 Nm, and a support cam can be used to offset the spring–torque profile. We characterized this device in [26] and found that the maximum assistance provided with a 15° offset was about 25 Nm. Therefore, in the dynamic condition the StreamEXO provides about twice as much assistance as the Laevo v2.56. This is relevant because it can help to understand how the exoskeletons’ performance scales when increasing the load. Figure 3 compares the different assistive profiles.

### 2.2. Lab Validation

**Figure 4:**
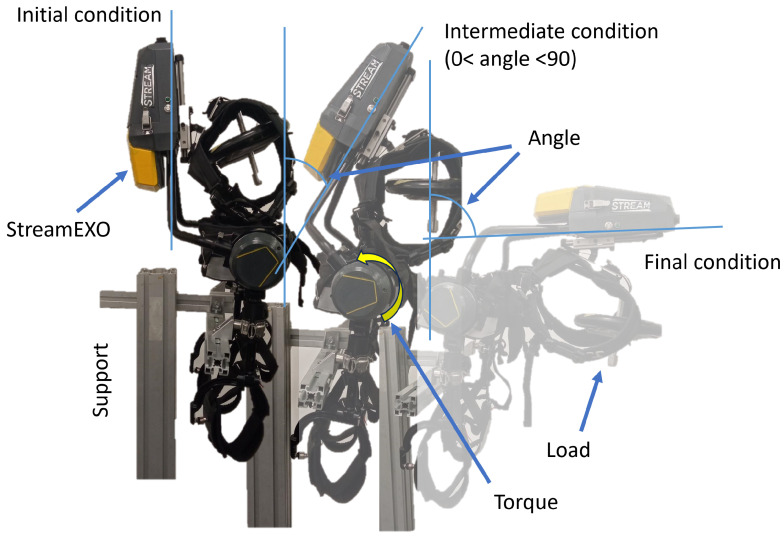
The system used for lab testing of the StreamEXO’s gravity compensation algorithm.

This section aims to evaluate the performances of the static assistive control strategy developed to reduce the gravitational weight of the wearer’s upper body. The test bench was set up as shown in Figure 4. The exoskeleton was mounted on a structure connecting the lower part of the exoskeleton’s frames. The upper structure was able to move freely as controlled by the actuators mounted just above the connection with the exoskeleton’s legs. The upper structure consisted of the exoskeleton’s frame, the shoulder straps, the electronics control module, and the battery. External loads were connected to the shoulder straps using a barbell that allowed stacking of up to four 5 kg disk weights. The test consisted of moving the exoskeleton by hand from its initial condition to the final condition, as shown in Figure 4. The inclination angle of the exoskeleton with respect to the vertical axes (θ) was measured. The test involved slowly changing the exoskeleton’s inclination from θ= 0° to θ= 90°. During this transition, a series of predefined posture angles [0°, 30°, 50°, 60°, 70°, 90°] were maintained for a few seconds. The test was repeated four times with different external loads of [5 kg, 10 kg, 15 kg, 20 kg].

The open-loop gravity compensation algorithm based on Equation (6) was correctly tuned to compensate for the external loads and generate the torque needed by the actuators to keep the exoskeleton structure consistently stable at the preset inclination angle. The real-time logs allowed us to record measured torque at the actuators, the inclination angle, and the acceleration components in the sagittal plane.

### 2.3. Gravity Compensation Control Strategy Evaluation

**Figure 5:**
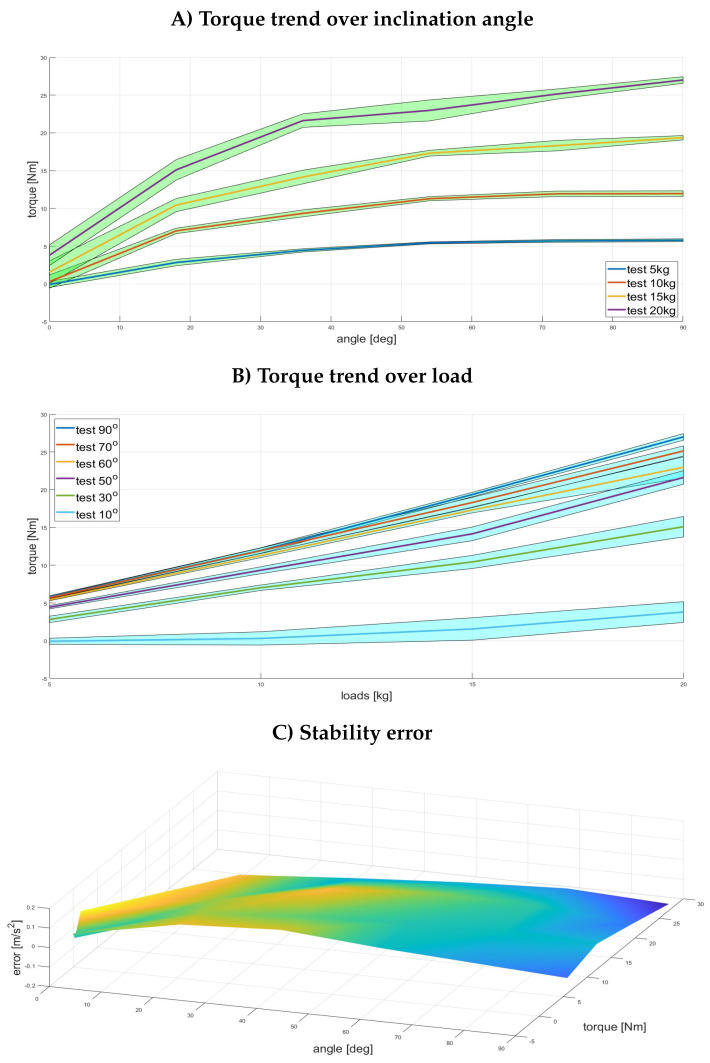
Results of the laboratory test conducted to assess the gravity compensation algorithm: (**A**) the torque trends as a function of the exoskeleton inclination; (**B**) the torque trends as a function of the external loads; and (**C**) the stability error measured as the vertical component of the instant exoskeleton acceleration mapped for the inclination angles and generated torque values due to the external loads.

The assessment was conducted on six different exoskeleton inclinations (θ∈ [0°, 30°, 50°, 60°, 70°, 90°]) and four externally applied loads ([5 kg, 10 kg, 15 kg, 20 kg]). Figure 5A shows the torque trends as a function of the inclination angle (θ) for the four external loads required to balance the exoskeleton. Figure 5B shows the torque-to-external load relationship for each steady inclination angle θ. Figure 5C shows the system’s stability for each of the conditions mentioned above. The stability was indirectly measured using the embedded IMU on the exoskeleton to measure sudden movements, vibrations, and sharp acceleration.

The results are displayed in Table 1, where the stability error for each of the six different angles is averaged. The absolute stability error was measured across all the weights and inclinations as $0.045\pm 0.124\;{m/s}^{2}$. These results demonstrate that the exoskeleton remained stable when controlled using the gravity compensation algorithm to counteract the weight applied to the upper exoskeleton’s structure. This is particularly evident between 30° and 70°, while the error slightly increases at the two extremities of the inclination angle range. Moreover, the control algorithm has been fully characterized up to 20 kg loads. These loads were placed higher than the upper body central gravity location (about 0.38 m from the motors instead of as in Equation (4): 0.18∗BH∼0.3). This forced the controller to generate higher torques than shown in Figure 3, where the assistive profile generated for the “typical” (1.75 m tall, 85 kg) worker reached about 39.3 Nm when using both motors (as shown in Figure 3).

## 3. Realistic Task Assessment: Experimental Protocol

**Figure 6:**
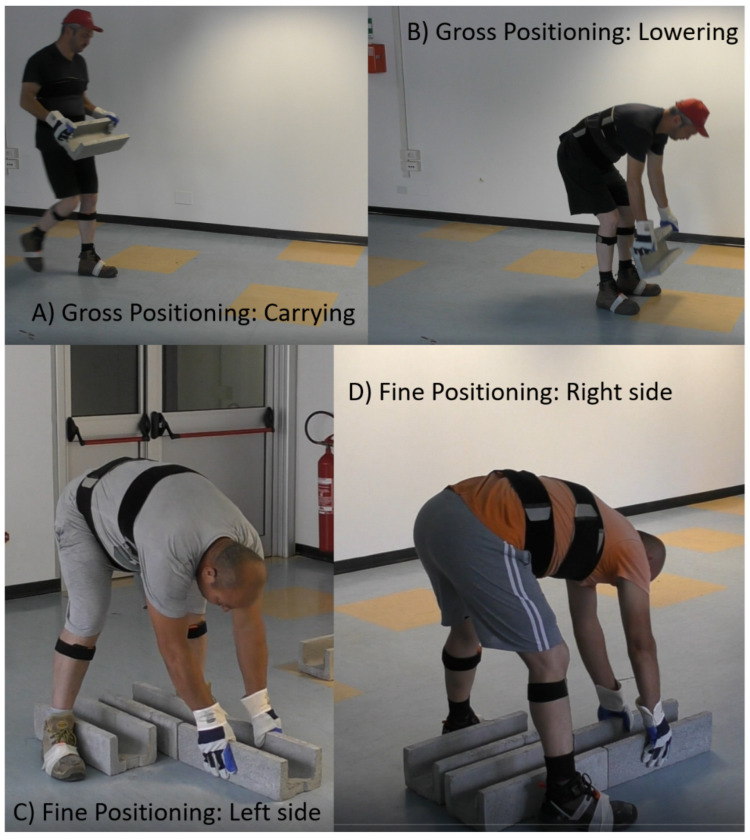
Images showing (**A**,**B**) carrying and lowering the cable duct during the “gross” positioning task and (**C**,**D**) the “fine” positioning of the cable duct on the left and right sides.

Ten male rail workers (age 45.4±9.5 years old; height 177.7±7.1 cm; weight 84.2±13.7 kg; average years of experience 11±11 years) were asked to take part in an experiment to simulate the two main activities performed during cable conduit replacement, namely, (i) gross positioning and (ii) and fine positioning [19]. The gross positioning task (Figure 6) involved lifting a 20 kg cable duct from ground level and carrying it with two hands for 2 m before lowering it again on to the ground. The workers then performed a 180° rotation and repeated the same sequence a further nine times, for a total of ten repetitions. The fine positioning of the cable duct is shown in Figure 6. This time, the workers were required to position themselves with their legs spread apart and bending forward. The workers lifted the cable conduit a few centimetres off the ground, moving it from the area close to the left leg to the right leg in order to align the conduits precisely. The workers remained in this static bent pose for 10 s and then stood up. This cycle was designed to maintain an average static task frequency similar to what was observed on-site. The activity was repeated ten times. Both the gross and fine positioning simulations were designed to mimic as closely as possible the actual work pace and postures that the workers adopted during real field tests. Considering the frequency, postures, and handled loads, these workers were exposed to an NIOSH Lifting Index of 1.73 and 1.50 for the gross and fine positioning tasks, respectively. These NIOSH values determine a moderate ergonomic risk for the tested MMH tasks. Therefore, it is extremely interesting to understand how the use of an exoskeleton can be beneficial for these working activities.

Finally, the gross and fine positioning tasks were performed by the ten workers under three different conditions, namely:

(1) noExo: activities performed without any type of exoskeleton
(2) laevo: activities performed with assistance provided by the passive Laevo v2.56 exoskeleton
(3) stream: activities performed with assistance provided by the StreamEXO

More details on the exoskeletons and their assistive strategies are reported in Section 2.1.

The experiments were approved by the Ethical Committee of Liguria (protocol reference number: CER Liguria 001/2019) and complied with the Helsinki Declaration. All of the subjects signed a consent form prior to participating and after a full explanation of the experimental procedure.

### 3.1. Data Collection

The objective of the lab simulation was to collect measurements that are typically considered too invasive or difficult to measure in the field, specifically, back muscle activation levels and kinematic data.

For the objective data measurements, we used the Xsens wearable motion tracking system to record full-body kinematics (MTw Awinda3D, Wireless Motion Tracker, Xsens Technologies B.V., Enschede, The Netherlands) at a sampling rate of 60 Hz, while back muscle activation levels were recorded bilaterally at 1 kHz using surface electromyography (sEMG) electrodes (BTS FREEEMG, BTS Bio-engineering, Garbagnate Milanese, Italy). These latter sensors were placed on the workers’ backs according to the SENIAM (http://www.seniam.org (accessed on 20 December 2023)) guidelines to measure the Erector Spinae Longissimus and the Erector Spinae Iliocostalis muscle activity. Before the start of each testing session, we measured the Maximum Voluntary Contraction (MVC) value for each back muscle [30].

### 3.2. Data Processing and Statistical Analysis

In this work we are primarily interested in understanding how oBSEs can assist workers with lifting tasks. Thus, although the workers had to perform a carrying task during the gross cable duct positioning, this is not considered in the following analysis. We extracted the average Erector Spinae median activation (ES median) for each worker, as this can be linked to the cumulative fatigue that a worker experiences [31], along with the peak values (ES peak). Higher values correlate with increased risk of developing MSD injuries. The ES median and peak values were computed by averaging the right and left side activation levels of the Erector Spinae Longissimus and the Erector Spinae Iliocostalis. These signals were band-pass filtered (35–350 Hz), smoothed, rectified, and subsequently normalized with respect to the MVC values.

As previusly mentioned in Section 2.1, the trunk inclination in the sagittal plane (θ) is an important parameter for estimating the assistance of the two oBSEs. For this reason, we calculated the average median trunk angle (Tmed) for each subject, as this can be used to reconstruct the assistance provided.

For statistical analysis, we applied a one-way repeated ANOVA analysis to all normally distributed data, considering the control strategy (**noExo**, **laevo**, and **stream**) as within-subject factors. We then used post hoc Bonferroni tests to compare the effects of the different control strategies for the metrics with a significant effect on the control strategy itself (*p*-value <0.05). The Bonferroni adjustment was 0.0167 and the number of degrees of freedom was 27. We used Matlab 2021b (The Mathworks, Natick, MA, USA) and R v3.3.3 (R Foundation for Statistical Computing, Vienna, Austria) software to perform the statistical analysis. Matlab 2021b was used to perform all of the data processing.

## 4. Results

Table 2 reports the results obtained for the two metrics under analysis (ESmed and Tmed) according to the activity (static or dynamic) and the test condition (**noExo**, **laevo**, **stream**). Bold values in the table report conditions for which we found a statistically significant difference. In particular, from the muscle activation analysis, the data were always statistically significant apart from the laevo condition during the dynamic test (*p*-value =0.138 for the median index, *p*-value =0.175 for the peak index). On the other hand, for Tmed only the StreamEXO did not show statistical significance on both tasks (*p*-value =0.093 for the dynamic task, *p*-value =0.231 for the static task).

**Figure 7:**
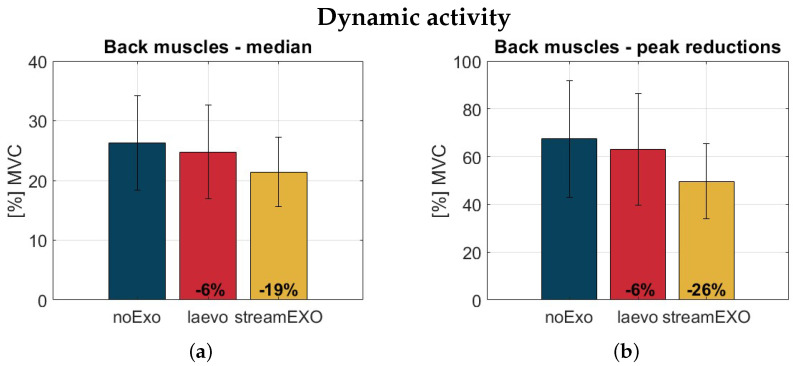
Bar plots of the data distributions for the median back muscle activation (**a**) and peak activation (**b**) during the dynamic activity for the three conditions under analysis: noExo (blue), laevo (red), and stream (yellow). The y-axis reports the ESmed values normalized with respect to the Maximum Voluntary Contraction value of each subject. The vertical segments are centered on top of the bars and are used to represent the standard deviation values.

**Figure 8:**
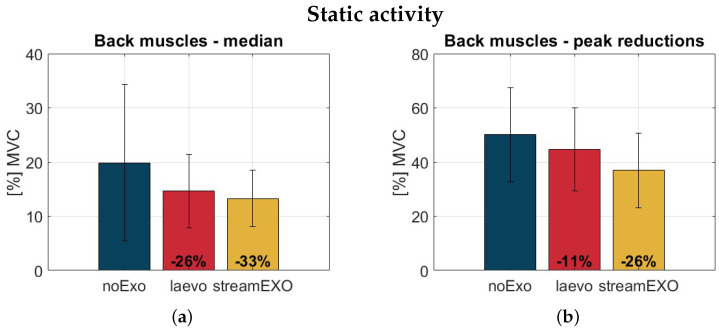
Bar plots of the data distributions for median back muscle activation (**a**) and peak activation (**b**) in the static activity for the three conditions under analysis: noExo (blue), laevo (red), and stream (yellow). The y-axis reports the ES values normalized with respect to the Maximum Voluntary Contraction value of each subject. The vertical segments are centered on top of the bars and are used to represent the standard deviation values.

Figure 7 and Figure 8 show bar plots of the data distributions for the median and peak values of back muscle activation during the dynamic and static tasks, respectively. The performance of the StreamExo shows different values of the median and peak indexes for both tasks. These values are all within the range of 19% to 33%. While the Laevo is seen to perform fairly well in the static task, this is not the case for the dynamic task. In particular, the StreamEXO generates a peak reduction of 26% in the dynamic task, compared to only a 6% reduction for the Laevo. The median is reduced by 19% for the StreamEXO, and again by 6% for the Laevo. In the static task, the median values are 26% for the Laevo and 33% for the StreamEXO, while the peak indexes show a reduction of 26% for the StreamEXO and 11% for the Laevo. Overall, for both the StreamEXO and the Laevo there is always an ES activation reduction; however, the StreamEXO generates a mean reduction more than double that of the Laevo (26% vs 12.25%) when averaging the four evaluated indexes (median and peak for the dynamic and static tasks). The biggest reduction (−33%) was obtained in the static activity when considering the median index. This benefit was achieved using the StreamEXO and the new control strategy (Section 2.3).

**Figure 9:**
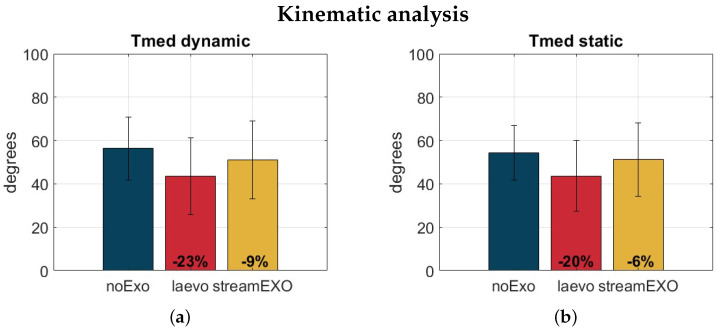
Bar plots of the data distributions for the median trunk inclination (Tmed) in the dynamic activity (**a**) and static activity (**b**) for the three conditions under analysis: noExo (blue), laevo (red), and streamEXO (yellow). Values are in degrees. The vertical segments are centered on top of the bars and are used to represent the standard deviation values.

Figure 9 shows the bar plots of the data distributions considering the median trunk inclination. Overall, it is interesting to note that the Tmed values are quite similar according to the task: about 55° for the noExo condition, 44° for the laevo condition, and 51° for the stream condition. These results suggest that using an oBSE may slightly alter the trunk angle, and consequently the user’s posture. For the StreamEXO, the posture adjustment was relatively small at 9% for the dynamic and 6% for the static test. For the Laevo, the change to posture was 23% and 20% for the dynamic and static tests, respectively. These values are relatively significant, and suggest that the passive system, which cannot be tailored to the tasks as effectively, may potentially introduce unexpected postural problems. This would, of course, need to be studied further in the future.

## 5. Discussion

Figure 7 and Figure 8 show that the usage of oBSEs, whether of passive or active type, can reduce activation in the back muscles. This represents a very positive benefit in both cases. The results for the StreamEXO are all statistically significant, while the Laevo shows significance only for the static task, as could be expected. This is an interesting trend that confirms the typical laboratory findings normally obtained when analyzing populations of students and using limited loads. Indeed, to the best of the authors’ knowledge this is the first study in which actual workers have been recruited to replicate their typical working activities in a laboratory setting, which allowed heavy loads (20 kg, representative of a true working environment) to be included in the experimental protocol. This extends the system for testing exoskeletons, bringing it closer to real-world validation.

Not surprisingly, back muscle activation for both dynamic and static tasks is reduced more with the active exoskeleton. This confirms the initial consideration based on the torque generated by the two compared systems. Indeed, as reported in Figure 3, the assistance provided by the StreamEXO is about twice that provided by the Laevo v2.56 exoskeleton. A similar proportion was found for the overall reduction values (Section 4). In addition, the new compensation strategy developed in this work outperforms the strategies presented in our previous work [26], further reducing muscle activation from 22% to 33%, suggesting that a dedicated biomechanical model-based algorithm is a winning recipe.

Another interesting outcome is that the active exoskeleton performed better in the dynamic activity (with the workers performing heavy manual labour) both in respect of assistance (reduced effort) and hindrance (posture/inclination similar to the **noExo** case). This reduced hindrance for the StreamEXO (a 9% reduction of Tmed) is possibly correlated with the actuation technology of the oBSEs under analysis. The Laevo v2.56 has fixed springs that increase in stiffness as the user bends, which may have discouraged the workers from bending the trunk. In contrast, the software-controlled motion and assistance of the StreamEXO aims to limit interfere with the workers’ motions. This effect was underlined during the static test, where the StreamEXO only slightly hindered the workers (a 6% Tmed reduction), while the Laevo reduced the trunk angle by 20%. We believe that this result is possible because of the new MB-Grav control strategy presented and validated in this work. This highlights that the assistance force generated in both control strategies, but in particular for the static case, manages to balance the person’s weight according to the trunk’s inclination without requiring extra compensation forces from the user. These strategies have been designed to balance the force component that supports the posture, leaving the user with the dynamic force component used to change the inclination of the trunk when necessary.

A further important consideration is the stability of the exoskeleton when worn. The StreamEXO takes advantage of straps on the thighs and shoulders to anchor the device, which is not present in the Laevo v2.56 design. In addition, both the thigh pads and trunk pad are free to move once fitted. As the Laevo v2.56 lacks these features, it is possible for the exoskeleton to move or rotate when maintaining a position for several seconds, thereby reducing its effectiveness.

Concerning the assistance provided by the StreamEXO, it is worth mentioning that in this study we derived a new strategy based on gravity compensation principles. To make for a better comparison, the parameters were not tuned to properly fit each subject and the average values of BW and BH were set for all the subjects. However, it is expected that personalized tuning will further improve the exoskeleton’s performance as well as the user experience. The human-in-the-loop approach can be used to optimize the control strategy coefficients based on subjective anthropometric data and performance dynamics [32,33,34].

## 6. Conclusions

This study evaluated the effectiveness of occupational back support exoskeletons (oBSEs) for railway workers during daily work activities performed in a laboratory setting. Ten male workers were asked to perform gross and fine positioning tasks using a concrete block weighing 20 kg while wearing no exoskeleton, a passive exoskeleton (Laevo v2.56), and an active exoskeleton (StreamEXO). In addition, this work presents the subsequent testing and validation of an open-loop model-based static task assistive strategy for the StreamExo that compensates for the gravity of the wearer’s upper body weight (MB-Grav strategy). An assessment was performed to analyze median and peak muscle activity for the dynamic and static tasks. Any hindrances arising from wearing either the active or passive exoskeletons were evaluated using possible restrictions in the median trunk flexion. The results showed that both passive and active exoskeletons reduced the metrics under analysis. In particular, the active exoskeleton (StreamEXO) provided more than double the assistance and negligible hindrance compared with the passive device (Laevo). In addition, the performance demonstrated by the biomechanical model-based MB-Grav strategy outperformed our previous assistive strategy based on trunk inclination. These findings provide valuable insights for the development and adoption of oBSEs in the workplace, suggesting that both passive and active exoskeletons have a role to play in reducing MSD, Further, they suggest that although active exoskeletons are more complex and possibly heavier, the development of tailored assistive algorithms can deliver very significant reductions in muscle activation with minimal impact on posture. Enhancement and development of these algorithms would seem to be a very real goal for future development.

Our future work will target the use of the control strategy developed in this work during on-site operational activities. In addition, a deep analysis of the EMG trend during the execution of this task will be carried out to evaluate how muscle activity varies in comparison to the baseline (**noExo** modality) in increasingly complex real-world work scenarios.

## Figures and Tables

**Table 1:** Average values and standard deviations of the stability error for the analyzed inclination angle (θ∈ [0°, 30°, 50°, 60°, 70°, 90°]) computed for all four loads ([5 kg, 10 kg, 15 kg, 20 kg]). Values are reported using the avg±std convention.

θ	Stability Error [m/s^2^]	Standard Deviation [m/s^2^]
0°	−0.003	0.149
30°	0.001	0.141
50°	−0.022	0.120
60°	−0.051	0.095
70°	−0.074	0.103
90°	−0.117	0.137

**Table 2:** Average values and standard deviations of the considered metrics (ESmed and Tmed) computed for all ten workers under the noExo, laevo, and stream conditions for the static and dynamic tasks. Values are reported using the avg ± std convention, while the reduction relative to the noExo mode is recorded in parentheses. In the laevo and stream conditions, the values in brackets represent the percent variation with respect to the noExo condition. Bold values indicate those distributions for which the difference was statistically significant.

		noExo	laevo	StreamEXO
**Dynamic**	ES median [%MVC]	26.31 ± 7.52	24.76 ± 7.46 (6%)	**21.41 ± 5.49 (19%)**
	ES peak [%MVC]	67.37 ± 23.15	63.02 ± 22.08 (6%)	**49.64 ± 14.87 (26%)**
	Tmed [deg]	56.36 ± 14.04	**43.62 ± 17.11 (23%)**	51.03 ± 17.37 (9%)
**Static**	ES median [%MVC]	19.85 ± 13.70	**14.66 ± 6.42 (26%)**	**13.28 ± 4.97 (33%)**
	ES peak [%MVC]	50.07 ± 16.46	**44.74 ± 14.56 (11%)**	**36.99 ± 13.06 (26%)**
	Tmed [deg]	54.30 ± 11.88	**43.71 ± 15.40 (20%)**	51.21 ± 15.9 (6%)

## Data Availability

The datasets generated during the current study are available from the corresponding author on reasonable request.
